# Fermented soy products: A review of bioactives for health from fermentation to functionality

**DOI:** 10.1111/1541-4337.70080

**Published:** 2024-12-15

**Authors:** Iskandar Azmy Harahap, Joanna Suliburska, Asli Can Karaca, Esra Capanoglu, Tuba Esatbeyoglu

**Affiliations:** ^1^ Department of Molecular Food Chemistry and Food Development, Institute of Food and One Health Gottfried Wilhelm Leibniz University Hannover Hannover Germany; ^2^ Department of Human Nutrition and Dietetics, Faculty of Food Science and Nutrition Poznan University of Life Sciences Poznan Poland; ^3^ Research Organization for Health National Research and Innovation Agency Bogor Indonesia; ^4^ Department of Food Engineering, Faculty of Chemical and Metallurgical Engineering Istanbul Technical University Istanbul Turkey

**Keywords:** bioactive compounds, fermentation process, gut microbiota interaction, nutritional enhancement, sustainable food production

## Abstract

The increasing prevalence of metabolic diseases and the global drive toward achieving Sustainable Development Goals (SDGs) underscore the need for sustainable, nutrient‐dense foods. Soybeans (*Glycine max*), a critical global crop, offer promising solutions; however, their predominant use as animal feed raises concerns regarding food security and environmental sustainability. Fermented soy products—including tempeh, natto, and miso—are rich in bioactive compounds such as peptides and isoflavones, which offer potential therapeutic effects and hold cultural and nutritional significance. These fermented products provide bioactive profiles with unique health‐promoting properties. This review critically examines the bioactive compounds generated through fermentation, focusing on their bioconversion pathways in the gastrointestinal tract and their metabolic implications for human health. Recent consumer demand for novel food ingredients with additional biological benefits has fueled research into advanced extraction techniques, enhancing the functional applications of bioactive compounds from these soy‐based products. This review further explores innovations in extraction methods that improve bioactive yield and sustainability, reinforcing the applicability of these compounds in health‐promoting food interventions. The originality of this review lies in its in‐depth exploration of the gastrointestinal bioconversion of fermented soy bioactive compounds alongside the latest sustainable extraction methods designed to optimize their use. Future research should aim to refine fermentation and extraction processes, investigate synergistic microbial interactions, and develop environmentally sustainable production methods. These efforts have the potential to position fermented soy products as essential contributors to global nutritional security and sustainable food systems, addressing both public health and environmental needs.

## 1 INTRODUCTION

The increasing prevalence of metabolic diseases such as obesity, diabetes, and cardiovascular disorders poses significant challenges to global health (Fava et al., 2019; Rhee, 2022; Vaishya et al., 2023). Concomitantly, achieving the United Nations Sustainable Development Goals (SDGs), particularly those related to zero hunger (SDG 2) and good health and well‐being (SDG 3), necessitates innovative solutions to food security and nutrition. Food loss and waste remain critical issues, with an estimated one third of food produced for human consumption lost or wasted globally, resulting in economic losses projected at $7.5 trillion, exacerbating the lack of nourished food, and undermining efforts to eradicate hunger (Blakeney, 2019; Khutornaya & Sergienko, 2021; Nicastro & Carillo, 2021). These challenges highlight the need for sustainable, nutrient‐dense food sources that can contribute to both human health and environmental sustainability.

Soybeans (*Glycine max*) represent a major global crop, with production exceeding 350 million metric tons annually (Dilawari et al., 2022; Ejaz et al., 2022; Guo et al., 2022). However, the majority of soybean production is destined for animal feed, with 85% of produced soybeans utilized to produce meals for this purpose. In contrast, only a small percentage, approximately 6%, is directly used as human food, predominantly in Asian countries (Alsanie, 2021). This trend not only impacts food security but also raises environmental concerns, as the conversion of soy to animal protein is less efficient than direct human consumption (Messina, 2022). Additionally, the widespread use of genetically modified organisms (GMOs) in soybean cultivation has sparked debates over environmental impacts and food safety, further complicating the landscape of soy production and utilization. In response to these concerns, in the European Union, efforts are being made to promote domestic production of protein‐rich crops, such as soybeans. This initiative aims to reduce the dependency ratio on protein imports, thereby enhancing food security and sustainability within the region (Debaeke et al., 2022; Rotundo et al., 2024).

In contrast to the Western focus on soy as animal feed, Asian countries have long recognized soy as a staple food, integral to their culinary traditions and nutritional practices (Qin et al., 2022). Traditional fermented soy products such as tempeh, natto, miso, and soy sauce are not only culinary staples but also hold cultural significance and health benefits. As the primary ingredient, soybeans contribute a rich source of protein, essential amino acids, and phytochemicals, positioning them as an invaluable raw material. The fermentation process, driven by various microorganisms, transforms soybeans into functional foods with an enriched profile of bioactive compounds, including isoflavones, peptides, and vitamins. These compounds have been associated with numerous health benefits, such as promoting gut health (Al‐Nakkash & Kubinski, 2020), improving bone health (Harahap et al., 2024), and modulating immune function (Harahap & Suliburska, 2021). Additionally, the production of fermented soy products supports sustainable food systems by utilizing environmentally friendly processing techniques and contributing to waste reduction.

Tempeh is a traditional Indonesian fermented soybean product characterized by its firm, cake‐like texture and distinct nutty flavor. It is produced through the fermentation of cooked soybeans with the mold *Rhizopus* spp., resulting in a product that is rich in protein, vitamins, and bioactive compounds (Rizzo, 2024; Romulo & Surya, 2021; Teoh et al., 2024). Natto, on the other hand, is a traditional Japanese food made from whole soybeans fermented with the bacterium *Bacillus subtilis* var. *natto*. Natto is known for its sticky texture, strong aroma, and distinctive flavor, and it is particularly rich in nattokinase, an enzyme with fibrinolytic properties, as well as vitamin K2 (Pinontoan et al., 2021). Miso is a fermented soybean paste originating from Japan, produced by fermenting soybeans with salt and the fungus *Aspergillus oryzae*. Miso is used as a seasoning and is noted for its umami flavor and high content of proteins, vitamins, and minerals (Abbas et al., 2022; Allwood et al., 2021; Yue et al., 2021). Soy sauce is a liquid condiment made from fermented soybeans, wheat, salt, and *A. oryzae* or *Aspergillus sojae* molds, widely used in Asian cuisine for its savory flavor (Diez‐Simon et al., 2020; Gao et al., 2023). It seems that fermentation enhances the nutritional profile of soy by increasing the bioavailability of nutrients and generating bioactive compounds with potential health‐promoting properties.

Tempeh has been consumed for centuries, especially in Southeast Asia, particularly in Indonesia, due to its rich nutritional profile and unique health benefits (Ahnan‐Winarno et al., 2021; Sjamsuridzal et al., 2021). This ancient food product has garnered modern scientific interest as a potential source of bioactive compounds with significant health implications (Teoh et al., 2024). The fermentation process involved in tempeh production not only enhances the digestibility of soybeans but also leads to the formation of bioactive peptides, isoflavones, and other beneficial compounds. As dietary habits shift toward more plant‐based and functional foods, understanding the health benefits of traditional products like tempeh becomes increasingly relevant (Rizzo, 2024).

The rationale for exploring tempeh bioactives lies in their substantial health benefits and nutritional significance. Tempeh is a powerhouse of essential nutrients, including high‐quality protein (Qin et al., 2022), vitamin B12 synthesized during fermentation (Teoh et al., 2024), and minerals (Rizzo, 2024). Moreover, the fermentation process enhances the bioavailability of these nutrients and introduces additional bioactive compounds with potential therapeutic effects. Studies have indicated that tempeh consumption may contribute to improved gut health, reduced cholesterol levels, and enhanced antioxidant capacity (Afifah et al., 2020; Kusuma et al., 2022; Lo et al., 2022; Surya et al., 2024). Given the rising prevalence of metabolic diseases and the global pursuit of sustainable, nutritious food sources, tempeh stands out as a promising candidate for health optimization.

In recent years, the demand for new food ingredients has significantly increased, not only for their nutritional value but also for their potential to offer additional biological benefits to consumers (Knorr & Augustin, 2021). The pursuit of novel food sources and bioactive compounds has driven research toward developing advanced extraction and purification techniques that enhance the functionality of these ingredients in food systems. As functional foods continue to gain popularity, the need for efficient methods to isolate and purify bioactive compounds from natural sources, such as soy‐based tempeh, becomes essential. Recent literature highlights several strategies for extracting bioactives from plant‐based sources, with applications ranging from new nutraceuticals to the improvement of existing formulations (e.g., capsaicin, gingerol, arabinoxylans, carminic acid, mangiferin, and natural sweeteners) (Castro‐Muñoz et al., 2024; Castro‐Muñoz, Correa‐Delgado, et al., 2022; Castro‐Muñoz, Gontarek‐Castro, et al., 2022; Ferreyra‐Suarez et al., 2024; Garza‐Cadena et al., 2023; Hernández‐Pinto et al., 2024). These studies exemplify the current trends in developing sustainable extraction techniques that are both efficient and environmentally friendly, paving the way for the integration of bioactive compounds into functional foods.

Current trends in the extraction of bioactives from fermented soy highlight a shift toward more efficient and environmentally friendly techniques. Techniques such as solvent extraction and supercritical fluid extraction (SFE) continue to be widely used, yet they often require extensive solvent use and longer extraction times. The advent of ultrasound‐assisted extraction (UAE) (Anaya‐Esparza et al., 2023), microwave‐assisted extraction (MAE) (Zuluaga et al., 2020), and cold plasma (CP) (Oner et al., 2023) has introduced more sustainable options that mitigate these drawbacks. These advanced techniques are increasingly recognized for their ability to efficiently extract bioactive compounds with minimal environmental impact.

Despite these advancements, a comprehensive synthesis of the various extraction methods and the biological implications of the extracted bioactives in fermented soybeans is lacking. The current review uniquely integrates the journey of tempeh from fermentation to its functional health benefits, distinguishing itself from previously published reviews in the field. The review by Ahnan‐Winarno et al. (2021) provides a comprehensive historical perspective on tempeh, summarizing 60 years of research on its health benefits, fermentation, safety, processing, and sustainability. However, it lacks a detailed discussion on modern extraction techniques and the bioconversion processes within the gastrointestinal tract. In contrast, the review by Cao et al. (2019) focuses on the bioactivities of soy‐based fermented foods, highlighting the functional properties and action mechanisms but does not thoroughly explore the extraction techniques or the complete bioconversion pathway. Lastly, recent reviews (Rizzo, 2024; Teoh et al., 2024) have highlighted the health benefits of soy‐based fermented foods broadly, including various products such as natto and miso, but do not delve deeply into sustainable extraction methods. Therefore, this review aims to bridge this research gap by providing a detailed overview of the exploration of various facets of fermented soy research, encompassing bioactive compounds, bioconversion processes within the gastrointestinal tract, and current trends in extracting bioactive compounds from fermented soybeans. Moreover, this review aims to consolidate current knowledge on the bioactive potential of fermented soy products, examining how fermentation influences their health‐promoting properties and exploring their role in sustainable dietary patterns. To the best of our knowledge, no previous review has thoroughly integrated these aspects, highlighting the need for a detailed evaluation of current extraction methodologies and their impact on the bioactivity of compounds derived from fermented soybeans. By integrating diverse areas of study, this review offers a novel perspective that is crucial for guiding future research and development efforts, ultimately maximizing the health benefits of tempeh in both traditional and modern dietary contexts.

## 2 OVERVIEW OF BIOACTIVES IN FERMENTED SOYBEAN

**FIGURE 1:**
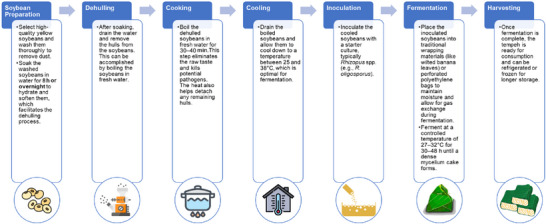
Preparation process of soy‐based tempeh.

The preparation process for soy‐based tempeh involves several steps, each with parameters optimized for effective fermentation. Figure 1 outlines the primary steps in soy‐based tempeh preparation, from initial soaking through fermentation. First, fresh soybeans are thoroughly washed and soaked in clean water for at least 8 h to hydrate the beans, making them easier to dehull and initiating natural acidification to inhibit unwanted microbial growth. After soaking, the beans are dehulled by rubbing or crushing, as intact hulls hinder mold growth essential for fermentation. The dehulled soybeans are then boiled for 30–40 min, which removes any raw flavors, eliminates potential pathogens, and facilitates further hull removal. Once boiled, the beans are drained and allowed to cool to between 25 and 38°C, the optimal temperature range for inoculation. The cooled beans are then mixed with a starter culture of *Rhizopus* spp., which requires even distribution to promote uniform mold growth. The inoculated soybeans are then packed in either banana leaves or perforated polyethylene bags, with each packaging method influencing the tempeh's final aroma and texture. Traditional banana leaves allow for better air exchange and impart a distinctive flavor, while polyethylene bags offer greater accessibility in various regions. Finally, the packed soybeans are incubated at 27–32°C for 30–48 h, during which the *Rhizopus* mycelium binds the beans into a solid cake. Proper management of variables such as temperature, humidity, and air exchange during this stage ensures successful fermentation and prevents contamination. This process may vary regionally, as local resources and traditional practices shape production methods, underscoring the adaptability of tempeh production (Ahnan‐Winarno et al., 2021; Teoh et al., 2024).

One of the major ways to enhance the bioactivity of components from soybeans is through fermentation. Fermentation plays a pivotal role in enhancing food quality and generating bioactive compounds beneficial for health. This process not only improves the bioavailability of nutrients but also produces bioactives with anti‐inflammatory, antioxidant, and antimicrobial properties, contributing to better gut health and reducing the risk of noncommunicable diseases like cardiovascular issues and type 2 diabetes. Additionally, leveraging fermentation to transform food waste into valuable byproducts helps minimize waste while enriching the nutritional profile of food products. This sustainable approach supports environmental conservation and promotes a circular economy in the food industry (Siddiqui et al., 2023).

These transformations result in enhanced bioactive profiles, making fermented soy products a valuable source of health‐promoting compounds. Moreover, fermented soybean products are notable for their rich content of bioactive compounds, which possess unique biological and chemical properties (Qiao et al., 2022). The fermentation process not only enhances the nutritional profile of soybeans but also generates a variety of bioactives that contribute to their health‐promoting effects (Liu et al., 2022). This section explores the key bioactives found in fermented soybeans and their potential health benefits.

### 2.1 Isoflavones

Isoflavones are phytoestrogenic and antioxidative compounds naturally present in soybeans and soy products. These compounds mimic the action of estrogen in the human body and exhibit potent antioxidant properties, which contribute to their health‐promoting effects (Arifin et al., 2021). Common isoflavones found in soybeans include daidzein, genistein, and glycitein, typically present in their glycoside forms (Křížová et al., 2019). Table 1 summarizes the key differences between isoflavone aglycones and glucosides, focusing on their frequency in natural products, solubility, absorption, biological effects, and production. Isoflavone aglycones, more prevalent in fermented foods, demonstrate higher bioavailability and bioactivity due to their lipid solubility, leading to rapid intestinal absorption and greater biological effects compared to glucosides. Isoflavone glucosides, widely present in various plants and foods, can be converted to aglycones through β‐glucosidases and demonstrate effective absorption, especially in their malonyl form (Hsiao et al., 2020). However, deconjugation is essential for their transport across the intestinal barrier. Table 2 provides an overview of the chemical structures of isoflavones and their glycosides, highlighting the structural differences between these compounds.

**TABLE 1:** Comparison of isoflavone aglycones and glucosides.

Characteristic	Isoflavone aglycones	Isoflavone glucosides	Reference
Frequency in natural products	More commonly found in fermented foods than in raw foods	Present in various plants and widely found in different kinds of food in variable amounts	Hsiao et al., 2020
Solubility	More lipid soluble, leading to increased bioavailability and greater bioactivity than glucosides	–	Hsiao et al., 2020
Absorption	Rapidly and efficiently absorbed into intestines	Malonyl isoflavone glucosides exhibited more effective absorption when compared to nonmalonyl isoflavone glucosides in terms of hydrolysis and absorption	Hsiao et al., 2020; Kim et al., 2022
Production from glucosides	The conversion of glucosides to aglycones is catalyzed by β‐glucosidases	–	Mól et al., 2023
Biological effects	Aglycones are more bioactive forms	Glucosides are less bioactive forms	Johnson et al., 2021
Conjugation	–	Due to their conjugation with glycosyl, flavonoid glycosides are generally not absorbed directly in the small intestine	Chen et al., 2022

**TABLE 2:** Chemical structures of isoflavones and their glucosides (Křížová et al., 2019).

	Aglycon			Glucoside			
	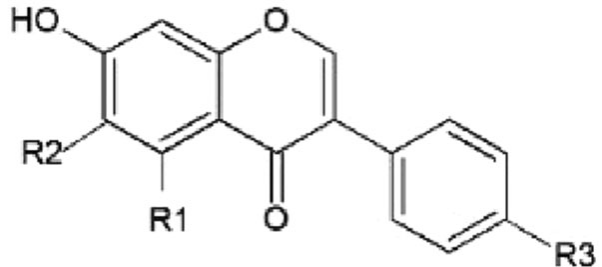			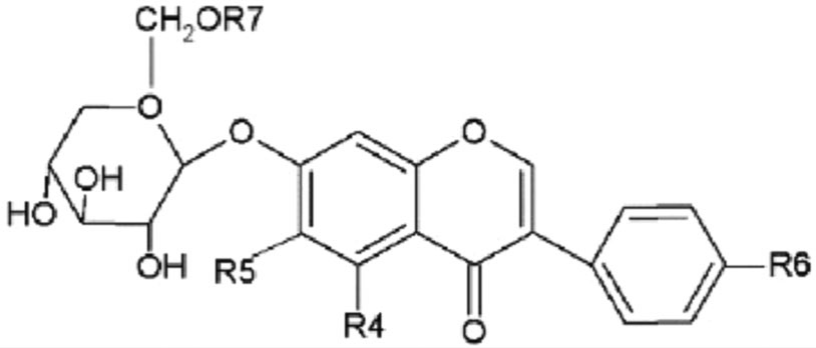			
	**R1**	**R2**	**R3**	**R4**	**R5**	**R6**	**R7**
Daidzein	H	H	OH				
Genistein	OH	H	OH				
Glycitein	H	OCH_3_	OH				
Formononetin	H	H	OCH_3_				
Biochanin A	OH	H	OCH_3_				
Daidzin				H	H	OH	H
Genistin				H	H	OH	H
Glycitin				H	OCH_3_	OH	H
Ononin				H	H	OCH_3_	H
Sissotrin				OH	H	OCH_3_	H
Acetyldaidzin				H	H	OH	COCH_3_
Acetylgenistin				OH	H	OH	COCH_3_
Acetylglycitin				H	OCH_3_	OH	COCH_3_
Malonyldaidzin				H	H	OH	COCH_2_COOH
Malonylgenistin				OH	H	OH	COCH_2_COOH
Malonylglycitin				H	OCH_3_	OH	COCH_2_COOH
Malonylononin				H	H	OCH_3_	COCH_2_COOH
Malonylsissotrin				OH	H	OCH_3_	COCH_2_COOH

The fermentation process used in tempeh making and natto fermentation significantly impacts the isoflavone content and composition of soybeans. During fermentation, microorganisms like *Rhizopus oligosporus* in tempeh or *B. subtilis* in natto metabolize the isoflavone glucosides (e.g., daidzin, genistin) into their aglycone forms (e.g., daidzein, genistein) (Lee et al., 2022). This conversion is facilitated by the action of β‐glucosidases, enzymes produced by the fermenting microbes, which hydrolyze the glycosidic bonds of isoflavone glucosides.

The biologically active aglycones are more readily absorbed in the human digestive tract compared to their glucoside counterparts. This transformation not only enhances the bioavailability of isoflavones but also increases their biological activity. The fermentation of soybeans thus leads to a substantial increase in the content of isoflavone aglycones, altering the isoflavone profile and enhancing their health benefits.

The identification and transformation of isoflavones during soybean fermentation underscore the importance of microbial processes in enhancing the nutritional and functional properties of soy‐based foods. The conversion of glycoside isoflavones to their aglycone forms by fermentation not only improves their bioavailability but also their bioefficacy. Among these bioactive compounds, 6,7,4′‐trihydroxyisoflavone (Factor 2), a major metabolite of daidzein, has demonstrated significant potential in various health applications. For instance, 6,7,4′‐trihydroxyisoflavone exhibits protective effects on neuronal cells, mitigating 6‐hydroxydopamine‐induced cell death in SH‐SY5Y human neuroblastoma cells (Ko, Kwon, et al., 2019). Additionally, another major metabolite of daidzein, 7,8,4′‐trihydroxyisoflavone, has been found to inhibit several pro‐apoptosis molecular factors, such as mitogen‐activated protein kinase and PI3K/Akt/GSK‐3β pathways (Ko et al., 2019). These findings highlight the multifaceted benefits of these isoflavone metabolites, making them valuable bioactive components in fermented soybean products with significant potential for improving human health.

The biosynthesis of isoflavones in soybeans involves several key metabolic pathways. Isoflavones are synthesized through the phenylpropanoid pathway, starting from phenylalanine. The initial step involves the conversion of phenylalanine to cinnamic acid by the enzyme phenylalanine ammonia‐lyase (PAL). This is followed by a series of enzymatic reactions involving chalcone synthase (CHS), chalcone isomerase (CHI), and isoflavone synthase (IFS), which lead to the production of isoflavone precursors such as naringenin and liquiritigenin (Sohn et al., 2021).

### 2.2 Bioactive peptides

Bioactive peptides, specifically those produced during tempeh fermentation, have been shown to exhibit various health benefits such as antioxidant, anti‐inflammatory, and antihypertensive effects (Tan et al., 2023). Tempeh fermentation, primarily mediated by *Rhizopus* species, plays a crucial role in the breakdown of soybean proteins into bioactive peptides. The production of bioactive peptides from soybeans involves the hydrolysis of soybean proteins during fermentation or enzymatic hydrolysis (Cruz‐Casas et al., 2021). This process is facilitated by the action of proteolytic enzymes either added externally or produced by microorganisms associated with fermentation. During fermentation, bacteria such as *B. subtilis* and lactic acid bacteria, along with fungi such as *Mucor* spp., *Aspergillus* spp., and *Rhizopus* spp., play a crucial role in breaking down soybean proteins into smaller peptide fragments. These microorganisms produce specific enzymes that catalyze the hydrolysis of soybean proteins (glycinin and β‐conglycinin), leading to the formation of bioactive peptides (Ketnawa & Ogawa, 2021). Each type of microorganism contributes to the production of different peptides, resulting in a diverse array of fermented products with unique bioactive profiles.

The biosynthesis of bioactive peptides in soybeans involves complex metabolic pathways that encompass proteolysis and peptidomic profiling during fermentation (Akbarian et al., 2022; Rizwan et al., 2023). Proteolysis, the breakdown of proteins into smaller peptides and amino acids, is a critical step in the generation of bioactive peptides. This process is mediated by proteolytic enzymes such as proteinases and peptidases, which are produced by fermenting microorganisms (Rizwan et al., 2023). During fermentation, specific enzymes cleave soybean proteins at designated sites, leading to the release of peptide fragments with potential health benefits. Peptidomic and transcriptomics profiling involves the identification and characterization of these peptides to understand their structure, function, and bioactivity. Advances in proteomics and peptidomics have enabled the detailed analysis of bioactive peptides, revealing their mechanisms of action and therapeutic potential (Rizwan et al., 2023).

### 2.3 Saponins

Saponins are triterpenoid glycosides that are abundantly found in soybean seeds. These compounds are known for their diverse biological activities, which include antiviral, antitumor, obesity prevention, and immune regulation effects. The presence of saponins in soybeans contributes significantly to their medicinal properties, making them a subject of interest in functional food research (Oleszek & Oleszek, 2020).

The microbial transformation can enhance the bioavailability and biological activity of saponins by producing various derivatives, such as ginsenoside, gypenoside, glycyrrhizin, saikosaponin, dioscin, timosaponin, astragaloside, and ardipusilloside (He et al., 2019). During fermentation, microorganisms metabolize saponins, leading to the production of compounds with improved solubility and enhanced biological activity. This transformation is critical as it increases the potential health benefits of soybean‐derived products. For example, fermentation can convert saponins into more bioactive forms, thereby enhancing their therapeutic effects and making them more readily absorbable by the human body (Qiao et al., 2022).

The fermentation process significantly transforms the saponin profile in soybeans, yielding products with enhanced bioactivity and reduced antinutritional factors. For example, specific strains like *B. subtilis* and *A. oryzae* have shown efficacy in solid‐state fermentation methods, where they synergistically improve the nutritional quality of soybean meal. Through two‐step solid‐state fermentation, these microbes not only increase protein content and antioxidant capacity but also markedly reduce antinutritional compounds such as saponins, phytic acid, and tannins (Suprayogi et al., 2022). Additionally, the specific structures and relative concentrations of saponins are notably altered by fermentation, impacting both the taste and health‐promoting properties of soy foods. Saponins are broadly classified as A and B, with the former often linked to a bitter taste and the latter associated with health benefits. During fermentation, group A saponins, which dominate in raw soybeans and contribute to bitterness, undergo partial or complete deacetylation, diminishing their intensity and enhancing palatability. This process not only mitigates the undesirable taste but also enriches fermented soy foods with bioactive compounds, positioning them as valuable functional foods for health‐conscious consumers (Chitisankul et al., 2021). These findings highlight the capacity of fermentation to enhance the nutritional quality and consumer appeal of soy foods, offering a strategic approach to optimize their health benefits and flavor profiles. Through targeted microbial action, fermentation not only improves the bioactivity of soybean components but also opens avenues for broader acceptance of soy‐based functional foods.

The biosynthesis of saponins in soybeans involves complex metabolic pathways that produce two main types of soyasaponins: group A saponins and 2,3‐dihydro‐2,5‐dihydroxy‐6‐methyl‐4H‐pyran‐4‐one (DDMP) saponins. The synthesis of these triterpenoid saponins is a multistep process, regulated by specific genes that control the production of key intermediates. One crucial gene in the biosynthesis of soyasaponins is the sg‐5 gene, which is responsible for the production of soyasapogenol A, a key component of group A saponins. Mutations in the sg‐5 gene can lead to metabolic switching, which results in the production of beneficial saponins over undesirable ones (Sundaramoorthy et al., 2019). Understanding the genetic regulation and metabolic pathways involved in saponin biosynthesis is essential for optimizing the fermentation process and improving the nutritional and pharmacological value of soybean products.

### 2.4 Phytosterols

**FIGURE 2:**
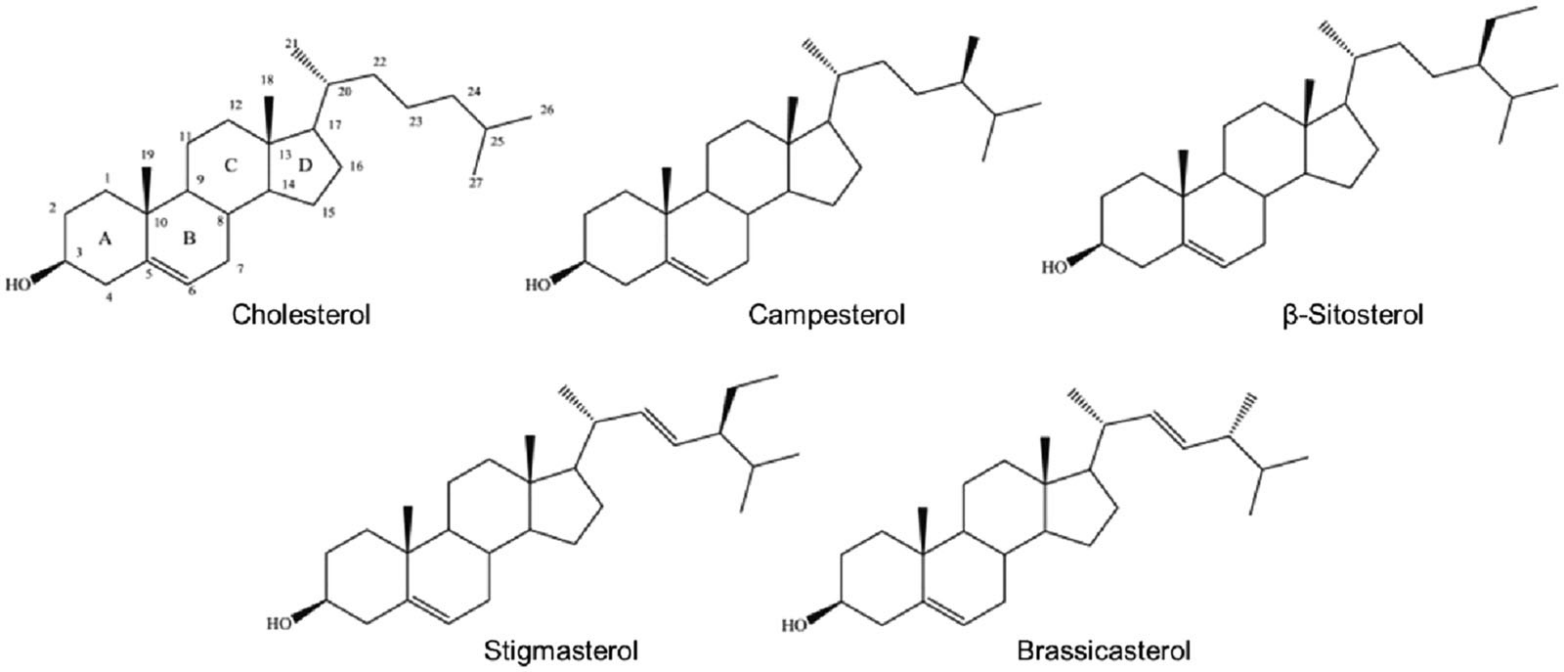
Chemical structures of cholesterol and common phytosterols.

Phytosterols are a class of bioactive compounds naturally found in soybeans, known for their cholesterol‐lowering properties and potential benefits in promoting cardiovascular health (Figure 2). These plant‐derived sterols can inhibit cholesterol absorption in the intestine, thereby reducing blood cholesterol levels and lowering the risk of cardiovascular diseases (Swallah et al., 2023).

Fermentation processes, widely used in the production of various soybean‐based foods, can significantly impact the levels and activity of phytosterols (Naresh et al., 2019). Fermentation has been shown to effectively increase the concentration of phytosterols in soybeans and related products. For example, solid‐state fermentation of soybeans with *R. oligosporus* RT‐3 has been found to enhance the antioxidant activity of soybeans. This enhancement is primarily due to the release of phenolic compounds during fermentation, which suggests additional nutraceutical benefits beyond the increased phytosterol content (Zhang, Wei, et al., 2022). The increase in antioxidant activity further underscores the potential health benefits of consuming fermented soybean products, making them valuable functional foods.

The biosynthesis of phytosterols in soybeans involves several key enzymes that are crucial for both their synthesis and conversion processes. The main enzymes participating in the biosynthetic pathway include cycloartenol synthase (CAS), sterol side chain reductase 2 (SSR2), sterol methyltransferase (SMT), Dwarf1 (DWF1), and cytochrome P450 710A (CYP710A), which are essential for the production of various phytosterols. Squalene synthase catalyzes the initial steps in the production of squalene, a key intermediate in sterol biosynthesis. Additionally, phospholipid acyltransferase (PSAT), acyl‐CoA acyltransferase (ASAT), and sterol glucosyltransferase (SGT) enzymes are responsible for converting free sterols into their conjugated forms. Among these enzymes, SMT and CYP710A are particularly significant for their role in modulating the composition of different types of phytosterols (Zhang et al., 2020).

### 2.5 Gamma‐aminobutyric acid

**FIGURE 3:**
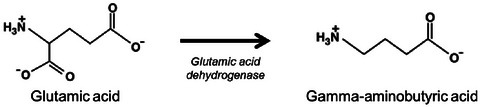
Biosynthesis of gamma‐aminobutyric acid (GABA) from glutamic acid.

Gamma‐aminobutyric acid (GABA) is a four‐carbon nonprotein amino acid that functions as a significant neurotransmitter in the central nervous system (Lee et al., 2023). It has gained considerable attention due to its health benefits, including lowering blood pressure, promoting relaxation, and improving mood (Hepsomali et al., 2020). Soybeans, particularly when fermented, can be a rich source of GABA (Sahab et al., 2020). GABA is produced through the action of glutamic acid decarboxylase, which facilitates the decarboxylation of l‐glutamic acid. l‐Glutamic acid is generated from α‐ketoglutarate in the tricarboxylic acid cycle by the enzyme glutamic acid dehydrogenase (Sahab et al., 2020). The chemical structures of glutamic acid and GABA are shown in Figure 3, illustrating the conversion process.

Fermentation processes involving specific strains and additives can substantially increase GABA content in soybeans. For example, another study demonstrated that the production of GABA in soybean milk by *Lactobacillus fermentum* resulted in a 1.7‐fold increase in GABA yield after optimizing the fermentation conditions (Rayavarapu et al., 2019). This indicates that fine‐tuning fermentation parameters can enhance GABA production in soybean‐based products. Furthermore, GABA‐producing *Lactobacillus brevis*, alone or co‐cultured with *Streptococcus thermophilus*, was used to ferment soy protein isolate medium with various sugars and monosodium glutamate. Results showed that co‐culturing enhanced sugar utilization, lactic acid production, GABA synthesis, and monosodium glutamate (MSG) reduction. The acidic environment from lactic acid production, rather than sugar utilization, significantly influenced GABA production by *L. brevis* (Xiao & Shah, 2021). These findings suggest that the selection of specific microbial strains and fermentation conditions can significantly impact the GABA content in soybean products, making fermentation a potent tool for increasing GABA levels.

### 2.6 Other bioactives

Soybeans are naturally rich in bioactive compounds, including vitamins, minerals, and fibers, which play essential roles in human nutrition. Vitamin B is crucial for growth and metabolism, serving as a co‐factor in various metabolic processes. Remarkably, tempeh contains vitamin B12, a vitamin typically lacking in plant‐based foods unless they are fermented. The production of vitamin B12 in tempeh is attributed not to fungi but to bacteria such as *Klebsiella pneumoniae* and *Citrobacter freundii*, which can be naturally present or added to the tempeh starter. This bacterial activity during fermentation is responsible for the increased levels of vitamin B12 in tempeh (Teoh et al., 2024). Additionally, minerals like calcium, magnesium, iron, and zinc are also abundant in soybeans (Bagale, 2021; Harahap et al., 2023; Montanha et al., 2023). The fermentation process, particularly by *Rhizopus*, was attributed to reducing phytate content and enhancing the presence of bioactive peptides, which likely facilitated greater mineral bioavailability (Erkan et al., 2020). These findings underscore the beneficial effect of fermentation in improving vitamin levels and mineral absorption from soy products like tempeh.

### 2.7 Modification of allergic proteins during fermentation

Understanding how fermentation affects the allergenic profiles of soy can further elucidate the overall impact of these processes on human health. Soybeans naturally contain various allergenic proteins and at least 30 other allergens, such as Gly m 1 to Gly m 8, P28, and P34, which can trigger allergic reactions in susceptible individuals (Pi et al., 2021). However, the fermentation process, particularly when carried out with specific microbial cultures like *Rhizopus* spp., *A. oryzae*, and *B. subtilis*, can modify or reduce these allergenic proteins (Rahim et al., 2023). Enzymatic activity during fermentation often leads to the hydrolysis of these proteins into smaller peptides, which are less likely to provoke allergic responses. For example, the fermentation of soybeans into tempeh and natto results in a significant reduction in allergenic potential compared to unfermented soybeans, as the microbial enzymes break down complex protein structures (Gopikrishna et al., 2021).

This reduction in antinutrients improves the overall digestibility and nutritional quality of fermented soy products. Thus, the fermentation of soybeans not only reduces allergenic and antinutrient factors but also enhances the nutritional value and safety of the resulting food products.

A comprehensive review by Pi et al. (2021) evaluated various processing techniques, including fermentation, for their effectiveness in reducing soybean allergenicity. The study concluded that fermentation, through enzymatic degradation and the action of fermentation strains and their metabolites, effectively reduces the allergenic potential of soybeans (Pi et al., 2021). Moreover, Yang et al. (2018) demonstrated that solid‐state fermentation with a mixture of *Lactobacillus casei*, yeast, and *B. subtilis* not only increased the total amino acid content but also degraded major allergenic proteins into low‐molecular‐weight polypeptides, thereby reducing the potential allergenicity of soybean meal. The study was supported by in vitro and in vivo assays, showing lower immunoglobulin E (IgE)‐binding capacity and milder allergic responses in animal models (Yang et al., 2018).

**FIGURE 4:**
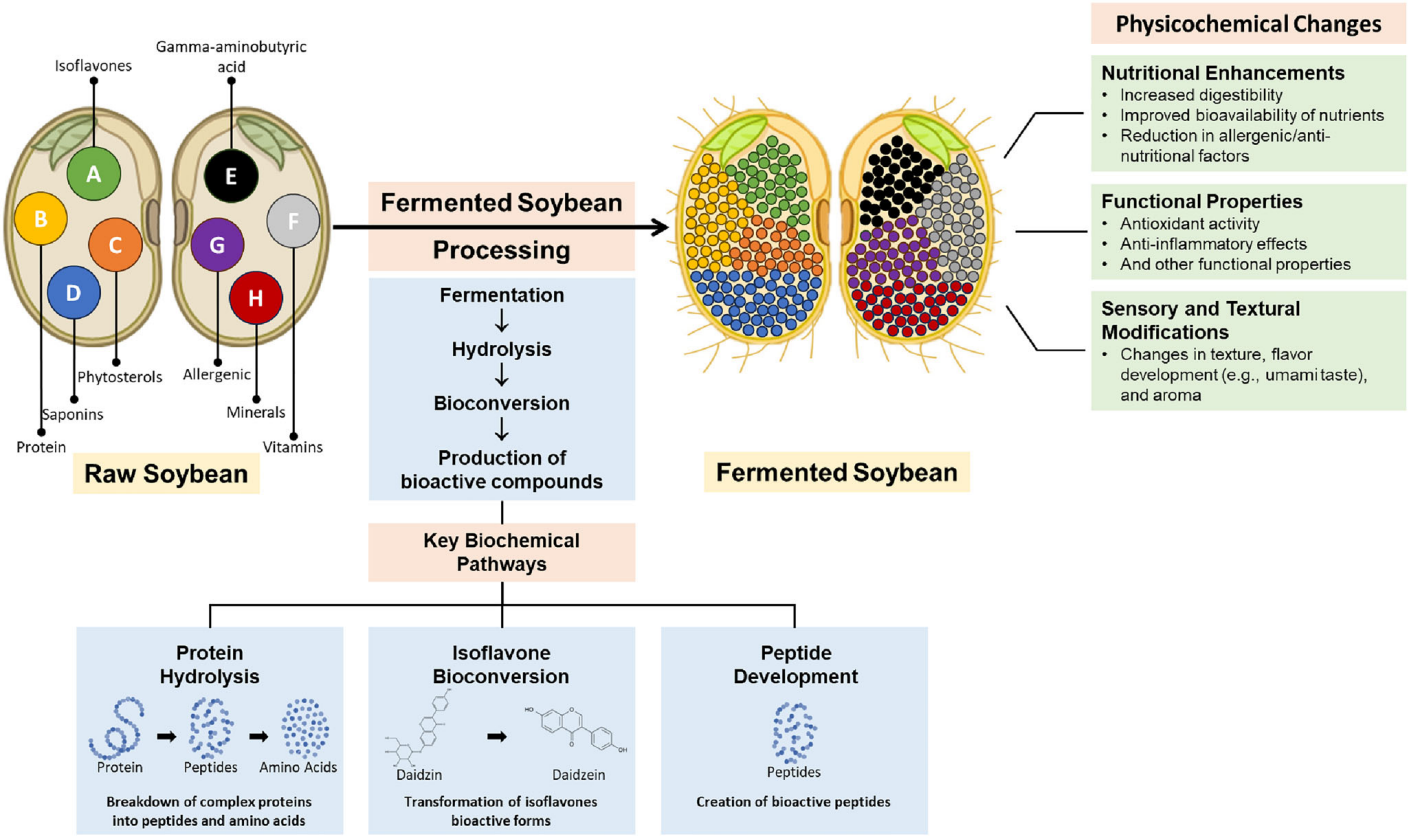
Biochemical pathways and physicochemical changes in soy‐based tempeh during fermentation.

Overall, these studies collectively underscore the transformative effects of fermentation on the allergenic and antinutrient properties of soy, offering a promising avenue for enhancing the nutritional and safety profile of soy‐based foods. The reduction in allergenic proteins and antinutrients through fermentation makes soy products more suitable for consumption, especially for individuals with sensitivities or allergies, and underscores the broader potential of fermentation in food processing and nutrition. Figure 4 illustrates the key biochemical pathways activated during the fermentation of soy‐based tempeh, including protein hydrolysis, isoflavone bioconversion, and bioactive peptide development. During fermentation, *Rhizopus* spp. enzymes break down complex proteins, resulting in smaller peptides and amino acids, which improve digestibility and bioavailability. Isoflavones undergo bioconversion, transforming into bioactive compounds such as daidzein and equol, which enhance antioxidant and anti‐inflammatory effects. Additionally, the formation of bioactive peptides contributes to antioxidant and antimicrobial properties, while fermentation also reduces anti‐nutritional factors like phytic acid, further increasing nutrient absorption. These processes yield notable sensory and textural modifications, enhancing tempeh's flavor, aroma, and texture, which are key to its acceptance as a functional food with potential health benefits, including gut health improvement, bone health support, and immune function modulation.

## 3 ENZYMES INVOLVED IN THE CONVERSION OF BIOACTIVES IN FERMENTED SOYBEAN

Fermentation of soybeans by microorganisms such as *Rhizopus* spp. is a complex biochemical process that enhances the nutritional value and bioactivity of soy‐based products. This process involves the action of various enzymes produced by both the soybeans and the fermenting microorganisms, which catalyze the breakdown of complex macromolecules into simpler, more bioavailable forms (An et al., 2023; Yao et al., 2024). Understanding the specific roles of these enzymes is crucial for optimizing the fermentation process and maximizing the health benefits of fermented soybean products.

The fermentation process of soy tempeh by *Rhizopus* spp. involves various enzymatic activities that are crucial for the conversion of soybeans into nutritious and bioactive‐rich food products. Key enzymes produced during this fermentation include protease, leucine aminopeptidase, carboxypeptidase, glutaminase, γ‐glutamyl transferase, and amylase (Kim et al., 2024). These enzymes catalyze the breakdown of complex proteins, carbohydrates, and other macromolecules in soybeans, resulting in the formation of simpler, more bioavailable nutrients. This enzymatic action leads to the production of a range of bioactive metabolites, including organic acids, antioxidants, and antimicrobial compounds. These metabolites not only enhance the nutritional value of tempeh but also contribute to its improved shelf life and health benefits (Kim et al., 2024).

The enzymatic activities lead to a significant increase in essential amino acids and peptides, which are easier for the body to absorb (Liu et al., 2022). Organic acids such as lactic and acetic acid lower the pH, inhibiting spoilage microorganisms and extending shelf life. Antioxidants neutralize harmful free radicals, and antimicrobial compounds provide natural preservation, ensuring the safety of tempeh (Liu et al., 2022).

Proteases, for example, break down soybean proteins into smaller peptides and free amino acids, enhancing digestibility and nutritional value (Teoh et al., 2024). Amylases hydrolyze starches into simple sugars, contributing to the unique flavor and texture of tempeh (Teoh et al., 2024). Lipases break down fats into fatty acids and glycerol, further utilized by fermenting microorganisms (Nur et al., 2020).

The enzymatic activities also enhance the probiotic potential of tempeh. *Rhizopus* spp. in soybean fermentation produces tempeh with high β‐glucan content and antibacterial activity against *Escherichia coli*, indicating its role in probiotic production during fermentation (Rizal et al., 2021; Teoh et al., 2024). Metabolic pathways during the solid‐state fermentation of soybeans with *R. oligosporus* involve biochemical reactions catalyzed by these enzymes. Protease activity hydrolyzes soy proteins into smaller peptides and amino acids, utilized by lactic acid bacteria (Ren & Li, 2022). Carboxypeptidases and leucine aminopeptidases further degrade peptides into amino acids, providing a rich nutrient source for probiotic bacteria growth (Nandan & Nampoothiri, 2020).

The biosynthesis pathways of isoflavones, saponins, and phytosterols are intricately linked with the fermentation process. Fermentation microorganisms, particularly the fungi involved, secrete specific enzymes such as β‐glucosidase that convert glycosidic isoflavones into their more bioactive aglycone forms (e.g., daidzein and genistein) (Mól et al., 2023). Similarly, saponins undergo structural modifications during fermentation, which may enhance their bioavailability and bioactivity (Qiao et al., 2022). Phytosterols, while not directly produced by fermentation, are influenced by the metabolic activities of the microbial community (Zhang et al., 2020). Exploring the specific roles of these enzymes in different fermentation conditions, such as varying temperature, pH, or microbial strains, could provide new insights into optimizing the production of bioactive compounds during tempeh fermentation. Future research should focus on how fermentation conditions can be tuned to enhance the biosynthesis and availability of these bioactives.

The mechanisms of fermentation in soy‐based products vary depending on the microbial species involved. For instance, tempeh is fermented with *Rhizopus* species, which promotes the breakdown of complex carbohydrates and proteins, leading to the formation of bioactive peptides and an increase in nutrient bioavailability (Rizzo, 2024; Romulo & Surya, 2021; Teoh et al., 2024). Natto, on the other hand, is produced by *B. subtilis*, which ferments soybeans into a sticky texture while generating unique bioactives like nattokinase (Pinontoan et al., 2021). In contrast, miso and soy sauce rely on *A. oryzae* and yeast to carry out fermentation, producing a complex mix of bioactive compounds and flavor‐enhancing metabolites (Diez‐Simon et al., 2020; Gao et al., 2023).

While lactic acid bacteria (*Lactobacillus* species) are frequently associated with the fermentation of dairy and vegetable products, their role in soy‐based fermentation is more limited. The lactic acid fermentation mechanisms do not apply directly to the primary soy products discussed in this review, as the key microbial agents in soy fermentation (*Rhizopus*, *B. subtilis*, *A. oryzae*) follow different pathways and enzymatic processes (Cao et al., 2019).

**FIGURE 5:**
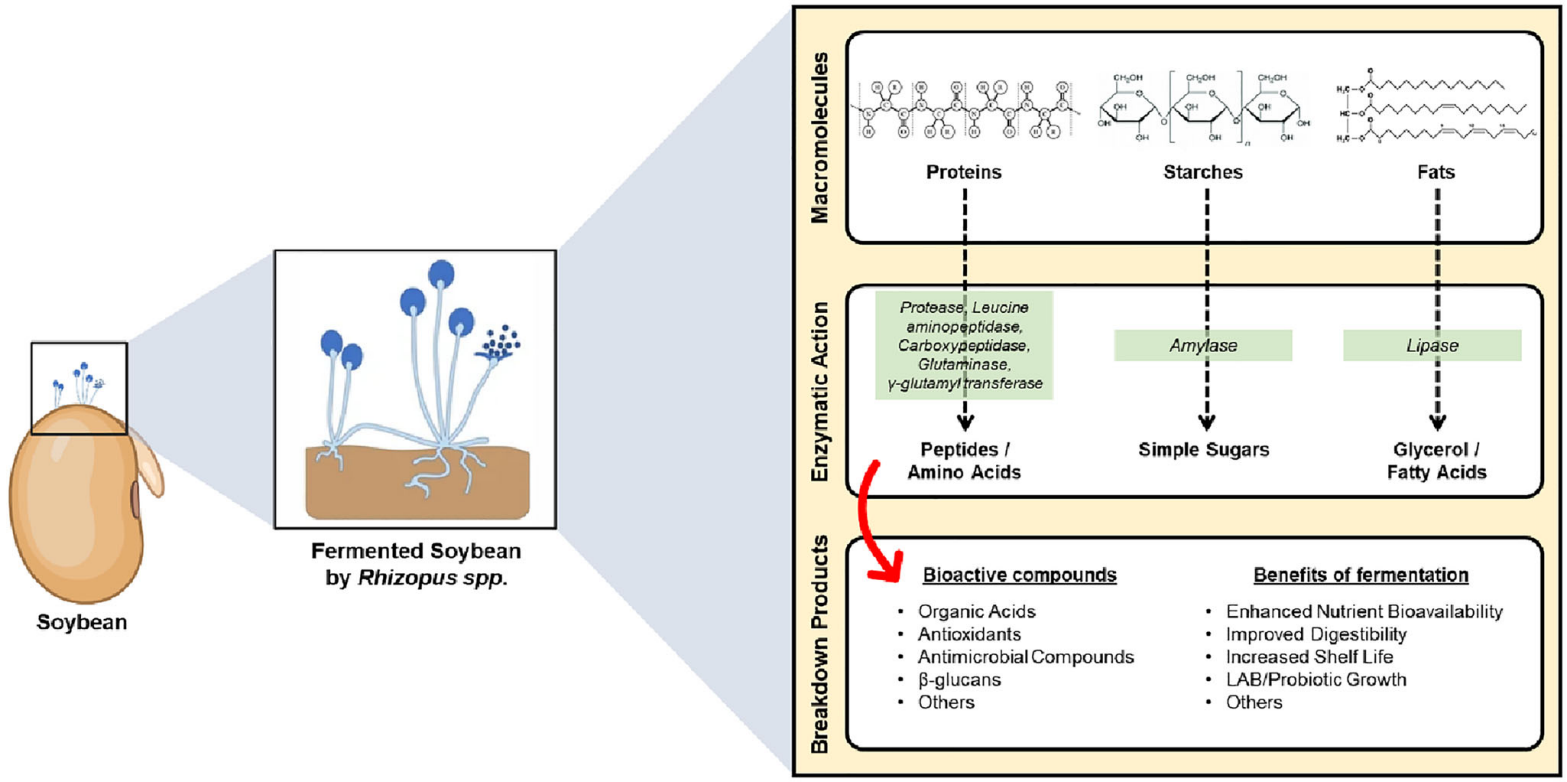
Enzymatic processes involved in the fermentation of soybeans by *Rhizopus* spp.

Figure 5 illustrates the enzymatic processes involved in the fermentation of soybeans by *Rhizopus* spp., highlighting key enzymes such as protease, leucine aminopeptidase, carboxypeptidase, glutaminase, γ‐glutamyl transferase, amylase, and lipase (Kim et al., 2024). These enzymes catalyze the breakdown of complex macromolecules in soybeans into simpler, more bioavailable forms, enhancing the nutritional value and bioactivity of tempeh. Proteases and peptidases contribute to the hydrolysis of proteins into peptides and amino acids, amylases break down starches into fermentable sugars, and lipases degrade fats into fatty acids and glycerol (Rizwan et al., 2023). The fermentation process leads to the generation of organic acids, antioxidants, and antimicrobial compounds, which collectively enhance the shelf life and health benefits of tempeh (Kim et al., 2024). Moreover, the enzymatic actions during fermentation promote the formation of bioactive metabolites and support the growth of lactic acid bacteria (Ketnawa & Ogawa, 2021), thereby significantly boosting the nutritional qualities of tempeh. These enzymatic activities also improve nutrient bioavailability and digestibility by breaking down complex proteins, carbohydrates, and fats into simpler forms that are more easily absorbed by the body (Wang, Fu, et al., 2022).

## 4 BIOCONVERSION OF BIOACTIVES IN FERMENTED SOYBEAN IN THE GASTROINTESTINAL TRACT

Understanding the bioconversion of bioactives in fermented soy during gastrointestinal digestion is essential for evaluating their health benefits. Fermentation not only alters the chemical composition of soybeans but also enhances the bioavailability of various bioactive compounds. Once consumed, these bioactives undergo further transformation through enzymatic and microbial activities in the digestive tract, which can significantly impact their efficacy and absorption (Cao et al., 2019). This phenomenon delves into the complex processes of bioconversion in the gastrointestinal tract, offering insights into the improved bioavailability and absorption of bioactives from fermented soy products.

The bioconversion of bioactives from fermented soy in the gastrointestinal tract is a complex process influenced by various factors. Firstly, the relationship between gut microbiota and bioactive production has been explored through studies on fermented soymilk. Consumption of fermented soymilk has been linked to the enhanced diversity and richness of gut microbiota, which in turn correlates with reduced systemic inflammation and the promotion of healthy microbial species (Madjirebaye et al., 2024). Furthermore, enzymatic and microbial fermentation processes represent another critical factor in enhancing the bioavailability of bioactives in soy. These processes synergistically convert glycosylated isoflavones into aglycones, elevate total phenolic content, and boost antioxidant capacity within soy extracts (Macedo et al., 2023). This enzymatic action, coupled with microbial metabolism, contributes significantly to the nutritional enhancement of fermented soy products. Besides, lactic acid bacteria fermentation is pivotal as well, influencing the microstructure and gastrointestinal digestibility of soy proteins. This fermentation alters the abundance of peptides and molecular interactions in the digestive tract, potentially enhancing the overall nutritional quality of soy products (Wang et al., 2022). The transformation of soy proteins by lactic acid bacteria not only affects digestion but also influences the bioactive profile available for absorption. Moreover, lactic fermentation of soy protein has been shown to impact human fecal microbiota composition. This modulation can affect protein digestion patterns and the distribution of intestinal microflora, highlighting its role in supporting intestinal health and function (Zhang et al., 2021). These changes underscore the intricate interplay between dietary fermentation and gut microbial ecology in human health.

In terms of gastrointestinal impact, fermentation significantly enhances soy protein digestibility. Specifically, lactic fermentation alters the digestibility profile of soy proteins, thereby increasing the bioaccessibility of proteins and bioactive compounds essential for human nutrition (Rui et al., 2019). Additionally, fermented soy products influence the structure and composition of the human gut microbiota. These products promote the growth of beneficial bacteria and restore levels of short‐chain fatty acids in the intestine, crucial factors for maintaining gut health and overall well‐being (Sun et al., 2024). The modulation of gut microbiota by fermented soy highlights its potential role in promoting a healthy gut environment conducive to overall health. In addition, each of these factors plays a significant role in determining the extent and efficiency of bioactive compound conversion and absorption.

The enzymes involved in each phase of the gastrointestinal tract also play a crucial role in the bioconversion of bioactives from fermented soy. In the oral phase, salivary enzymes initiate the breakdown of carbohydrates, setting the stage for further digestion. As the food bolus moves into the gastric phase, pepsin and stomach acids continue to break down proteins and other macromolecules. Lactic acid fermentation affects the digestibility rate of soy proteins, especially at the initial phases of gastric and duodenal digestion, thereby reducing the exposure of intact epitopes in the duodenum (Huang et al., 2020). The intestinal phase, particularly in the small intestine, involves a complex interplay of pancreatic enzymes and bile salts, which further hydrolyze proteins, lipids, and carbohydrates into absorbable units. Probiotic fermented soymilk has been shown to influence the activities of intestinal digestive enzymes, such as amylase, protease, and lipase, indicating the potential of fermented soy to modulate enzyme activity in the gastrointestinal tract (Huang & Wang, 2023). Each phase contributes to the progressive breakdown and transformation of bioactive compounds, enhancing their absorption and bioavailability.

In humans, the metabolic pathways of daidzein follow a somewhat similar but distinct course compared to rodents. After consumption, daidzein is metabolized primarily into *O*‐desmethylangolensin and equol. Equol, in particular, is recognized as the most active metabolite of soybean isoflavones due to its potent antioxidant and estrogenic activities (Ortiz & Manta, 2024). The conversion of daidzein to equol and other metabolites involves a series of enzymatic reactions, with glucuronidation and sulfation being predominant pathways (Wang et al., 2022).

Equol production varies significantly among individuals, with only about 20%–30% of the global population classified as “equol producers” (Li, 2019; Ortiz & Manta, 2024). An individual's ability to produce equol is determined by various factors, including the type and processing methods of soy foods, the presence of specific equol‐producing bacteria in the gut, and dietary patterns (Gong et al., 2023). Soy isoflavones are mainly found as glycosides, and their conversion into aglycones enhances bioavailability, which can be affected by how soy products are processed. For instance, steaming retains isoflavones, whereas soaking and alkali treatments can lead to significant losses. Lactose malabsorption may increase equol production by altering gut flora and enhancing the availability of isoflavones for conversion. Additionally, diets rich in carbohydrates, hydrogen, and short‐chain fatty acids such as propionate and butyrate tend to promote equol production, while high‐fat diets may inhibit it. Other internal factors, including genetic background, age, gender, and gut health, along with external factors like the source and form of soy isoflavones, significantly influence equol production (Gong et al., 2023). In addition to the bioconversion of bioactives in fermented soy during gastrointestinal digestion, the fermentation process itself plays a crucial role in enhancing the nutritional and health benefits of soy products.

## 5 ISOLATION OF BIOACTIVE COMPOUNDS BY EXTRACTION

Extracting bioactive compounds from fermented soybean products requires the application of effective and efficient extraction techniques to maximize yield and bioactivity. Traditional methods, including Soxhlet extraction, heat reflux, and maceration, have been widely used due to their simplicity and reliability. However, these methods often involve prolonged extraction times, high energy consumption, and the use of large volumes of organic solvents, which may not be environmentally friendly or cost‐effective (Barão et al., 2024; Chuo et al., 2022; Jha & Sit, 2022; Moreira et al., 2019). In response to these limitations, researchers have explored various innovative extraction technologies that offer significant advantages over conventional methods.

In contrast, innovative extraction methods have emerged as superior alternatives, offering enhanced efficiency, reduced environmental impact, and better preservation of bioactive compounds. Innovative processes such as UAE are increasingly being applied in the processing and extraction of bioactive compounds. UAE utilizes the principle of cavitation, which generates reactive species and enhances the physicochemical properties of food items. This technique not only improves the extraction efficiency but also modifies the structural and functional properties of the extracted compounds (Castro‐Muñoz et al., 2023). For instance, hydrodynamic cavitation has been shown to significantly alter the physicochemical properties of food ingredients, such as improving water‐holding capacity and solubility. Additionally, high‐pressure processing (HPP) has been demonstrated to impact the bioactive compounds in milk, affecting the behavior of proteins and other components (Siddiqui et al., 2024). These advanced techniques, including high hydrostatic pressure, UAE, and MAE, are increasingly preferred for their ability to yield higher extraction rates, use fewer solvents, and maintain the integrity of thermosensitive bioactives. Table 3 provides an overview of various conventional and innovative extraction techniques used to isolate bioactive compounds from plant materials. Each technique is described with its advantages, disadvantages, and general applications across a range of plant‐based bioactive extractions. It highlights the benefits and drawbacks of each approach, providing insights into their effectiveness and suitability for preserving bioactive compounds.

**TABLE 3:** Comparison of conventional and innovative extraction techniques.

**Extraction technique**	**Description**	**Advantages**	**Disadvantages**	**Reference**

Conventional techniques				
Soxhlet extraction	Continuous solvent extraction technique where the solvent repeatedly flows over the sample, dissolving the desired compounds	Efficient for a wide range of compounds, well‐established, and simple to use	Time‐consuming, high solvent usage, potential degradation of thermosensitive compounds due to prolonged heating	Torres‐Rodriguez et al., 2024; Zaid et al., 2023
Heat reflux extraction	Involves boiling the solvent and condensing the vapors back into the mixture, maintaining a constant extraction temperature	Simple setup, effective for various compounds	Extended extraction time, high energy consumption, and risk of degrading heat‐sensitive compounds	Kampwerth et al., 2020; Solomin et al., 2024
Maceration	Soaking the plant material in a solvent at room temperature for an extended period to dissolve the bioactive compounds	Simple and cost‐effective, no need for specialized equipment	Prolonged extraction time, lower efficiency, and potential degradation of bioactives	Barão et al., 2024; Lakshmanan, 2022

Innovative techniques				
High hydrostatic pressure (HHP)	Uses extreme pressure to disrupt cell walls and enhance the release of intracellular compounds without applying high temperatures	Preserves thermosensitive compounds, shorter extraction times, and lower solvent usage	Requires specialized equipment	Gómez‐Maqueo et al., 2020; Zemmyo et al., 2020
Ultrasound‐assisted extraction (UAE)	Employs ultrasonic waves to create cavitation bubbles that disrupt cell structures, facilitating the release of bioactives	Higher yields, shorter extraction times, reduced solvent usage, effective at lower temperatures	Equipment costs, potential for localized heating	Berhow et al., 2020; Laurora et al., 2020
Microwave‐assisted extraction (MAE)	Uses microwave energy to rapidly heat the solvent and plant matrix, leading to faster extraction and reduced solvent consumption	Fast extraction, lower solvent use, effective for thermosensitive compounds	Risk of uneven heating, requires microwave‐specific equipment	Ferrara et al., 2023; Nithya et al., 2023
Cold plasma extraction (CP)	Nonthermal technique that generates reactive species to enhance the extraction of bioactives without significant heating	Preserves heat‐sensitive compounds, environmentally friendly, minimal solvent use	High initial setup costs, technology still being optimized for wide‐scale application	Puente‐Díaz, 2024; Xi et al., 2024
Pulsed electric field (PEF)	Uses short, high‐voltage pulses to permeabilize cell membranes, allowing for the efficient release of intracellular compounds	Short processing times, energy‐efficient, preserves bioactivity	Requires specialized equipment	Domínguez et al., 2019
Supercritical fluid extraction (SFE)	Employs supercritical CO_2_ to extract nonpolar compounds with minimal solvent residues and high purity	High extraction efficiency, environmentally friendly, no solvent residue, suitable for thermosensitive bioactives	High operational costs, requires specialized equipment, limited to nonpolar compounds	Aili et al., 2024; Vinitha et al., 2022

While the table above outlines the comparative advantages of both conventional and innovative extraction techniques, it is essential to critically examine their applicability in the context of bioactive compound extraction from fermented soybeans. The composition and physical structure of the soybean matrix present specific challenges to extraction techniques. Soybean processing produces a substantial residue fraction, primarily composed of fibers and insoluble components. Managing this residue can be challenging, requiring additional processing steps for disposal or valorization. Furthermore, the presence of fibers can hinder downstream processing and reduce extraction efficiency (Gonzalez et al., 2023). In addition, the high moisture content of soybeans impacts extraction efficiency (Colletti et al., 2020). An overview of the extraction methods for bioactive compounds from fermented soy can be seen in Table 4.

**TABLE 4:** Overview of extraction methods for bioactive compounds from fermented soy products.

Food matrix	Bioactive compounds	Extraction method	Parameters	Result	Reference
Fermented soy sauce	Aroma‐active compounds (malty, alcoholic, fruity, floral, caramel‐like, smoky, sour, other)	High hydrostatic pressure	Pressure: 400 MPa Time: 30 min	Increased aroma‐active compounds: sour (19%), malty (37%), floral (37%), caramel‐like (49%), and other aromas (118%)	Zhang, Zhang et al., 2022
Low‐salt fermented soy sauce	Microbial community (*Staphylococcus*, *Bacillus*, *Kosakonia*, *Enterobacter*, *Enterococcus*, *Pseudocitrobacter*, *Lactobacillus*, *Eschericia*, *Shigella*, *Listeria*, *Cutibacterium*, other)	High hydrostatic pressure	Pressure: 300 MPa Temperature: 4°C Time: 5 min	Decreased bacterial abundance and increased bacterial diversity	Lai et al., 2022
Fermented soy sauce	Microbial diversity and volatile compounds	High hydrostatic pressure	Atmospheric pressure	Decreased fungal community diversity, decreased total volatiles, and increased organic acids and free amino acids	Liang et al., 2019
Fermented soybean meal	Peptides and proteins	Ultrasonic‐assisted extraction	Power density: 0.08 W/mL Frequency: 33 kHz Time: 1 h	Peptides: ↑ 31.27% and soluble proteins: ↑ 18.79%, compared to the control	Ruan et al., 2020
Fermented tempeh edamame	β‐Glucosidase enzyme	Ultrasonic‐assisted extraction	Frequency: 40 kHz Time: 30 min	β‐Glucosidase enzyme 15.51% higher than conventional method	Wibisono & Warsito, 2021
Fermented tempeh—germinated soy	Protein concentrate	Ultrasonic‐assisted extraction	Time: 20 min Temperature: Ambient	Germinated soy tempeh with 72.8% higher protein concentrate than nongerminated soy tempeh	Prayudani et al., 2023
Fermented okara soy	Soluble dietary fiber	Ultrasonic‐assisted extraction	Frequency: 40 kHz Power: 200 W Time: 25 min	Soluble dietary fiber three times higher than in control samples	Xie et al., 2024
Fermented soybean	Phenolic content	Supercritical extraction	Pressure: 25 MPa Temperature: 80°C	Phenolic compound content: 1391.9 µg GAE/g dry weight Yield: 42.87%	do Prado et al., 2022
Fermented soybean	Fatty acid composition and minor active components	Supercritical fluid extraction	Pressure: 30 MPa Temperature: 35°C	Unfermented Free fatty acids: 94.91 ± 0.71% Vitamin E: 159.96 ± 1.15 mg/100 g Phytosterols: 569.68 ± 3.55 mg/100 g Total flavonoids: 1.39 ± 0.02 mg/100 g Total polyphenols: 17.54 ± 0.31 mg/100 g Fermented Free fatty acids: 94.60 ± 0.30% Vitamin E: 162.31 ± 1.87 mg/100 g Phytosterols: 654.89 ± 20.09 mg/100 g Total flavonoids: 9.85 ± 0.90 mg/100 g Total polyphenols: 50.64 ± 4.44 mg/100 g	Xia et al., 2022

Table 4 illustrates that UAE and SFE have emerged as popular methods for extracting bioactives from fermented soybeans. In a study on the fatty acid composition and minor active components of fermented soybean, SFE was conducted at a pressure of 30 MPa and a temperature of 35°C (Xia et al., 2022). The results revealed substantial changes in the bioactive content of soybeans following fermentation. For example, phytosterol levels increased markedly from 569.68 mg/100 g in unfermented soybeans to 654.89 mg/100 g in fermented soybeans. The total flavonoid content experienced a notable enhancement, rising from 1.39 to 9.85 mg/100 g, while total polyphenols increased from 17.54 to 50.64 mg/100 g (Xia et al., 2022). These changes suggest that fermentation can significantly enrich the bioactive profile of soybeans, making fermented products potentially more beneficial in terms of antioxidant and health‐promoting compounds.

The increase in bioactive compounds due to fermentation can be attributed to the breakdown of complex molecules into simpler, more extractable forms. For example, the phytosterol content showed a notable increase of 14.96%, which is significant for its cholesterol‐lowering effects and other health benefits. The most remarkable increases were observed in the total flavonoid and polyphenol contents, with increases of 609% and 189%, respectively. This significant enhancement is likely due to the fermentation process, which involves microbial activity that enhances the extraction and conversion of these compounds. Additionally, fermentation leads to the formation of beneficial lipid fractions, such as free fatty acids and diacylglycerols, which not only contribute to the increased bioactive content but also protect cells from oxidative stress and lipid peroxidation, as evidenced by their ability to inhibit ferroptosis in PC12 cells (Xia et al., 2022). These compounds are known for their strong antioxidant activities, which can protect against various chronic diseases. The increase in these compounds suggests that fermentation can significantly enhance the health‐promoting properties of soybeans.

The data highlight the effectiveness of SFE in extracting bioactives from soybeans, particularly after fermentation. The increase in bioactive compounds such as vitamin E, phytosterols, flavonoids, and polyphenols emphasizes the dual role of fermentation and advanced extraction techniques in maximizing the nutritional value of soy‐based products. This enhancement is crucial for developing functional foods with improved health benefits. Furthermore, the substantial increases in flavonoids and polyphenols postfermentation underscore the potential of fermented soybeans in functional food applications. These findings suggest that incorporating fermented soybeans into the diet could offer enhanced antioxidant protection and other health benefits compared to unfermented soybeans. The increase in these bioactives also points to the potential for developing new fermented soy‐based products with targeted health benefits, leveraging the improved bioactive profile achieved through fermentation and advanced extraction methods.

In addition to the benefits of emerging technologies, such as nonthermal methods (e.g., HPP, ultraviolet treatment, pulsed electric fields, ultrasonication, CP, and supercritical carbon dioxide), it is critical to consider potential safety risks. These recent advancements in food processing have significantly contributed to enhancing food safety and quality. These methods are known for their energy efficiency, minimal processing impact, and ability to retain the nutritional and sensory properties of food. However, it is crucial to consider the potential risks associated with these technologies. For example, while nonthermal processes avoid high temperatures, they may induce chemical changes within the food matrix, leading to the formation of byproducts that have not been thoroughly evaluated for long‐term safety. Additionally, incomplete microbial inactivation poses a potential risk for microbial resistance in certain cases, raising food safety concerns (Jadhav & Choudhary, 2024).

## 6 CHALLENGES AND FUTURE DIRECTIONS

Despite the increasing interest in the bioactive compounds found in fermented soy products like tempeh, several key challenges impede the progress of research and development in this field. One of the primary obstacles is limited consumer acceptance, especially for bioactive‐enriched products. Although bioactives present in tempeh, such as isoflavones and peptides, have documented health benefits, the general public's understanding and acceptance of these functional compounds remain limited (Tso et al., 2021). This limitation poses a challenge to expanding tempeh's popularity beyond traditional markets in Southeast Asia and establishing it as a widely accepted functional food.

Another significant challenge is the standardization of fermentation processes to consistently produce tempeh with stable and effective bioactive profiles. The variability in microbial strains, fermentation conditions, and soy substrates affects the synthesis and stability of bioactive compounds, such as antioxidants and anti‐inflammatory agents, leading to inconsistent health outcomes in studies and consumer products (Tamang et al., 2022). This inconsistency presents challenges for reproducibility in research and the development of standardized products with predictable health benefits. Moreover, understanding the bioactive interactions within the complex food matrix and during digestion requires more extensive and controlled studies to confirm health claims.

Addressing these challenges requires innovative strategies and a multidisciplinary approach. One promising direction is the enhancement of tempeh's functional applications, particularly through precise control over bioactive production in the fermentation process. Tempeh's potential as a plant‐based source of bioactive compounds offers opportunities for health‐conscious consumers who seek both nutritional and functional benefits (Imran & Liyan, 2023). Advancements in biotechnological tools and fermentation techniques are essential for overcoming the standardization challenge. Employing precision fermentation (Terefe, 2022), using well‐characterized microbial strains known for specific bioactive production (Zahn, 2017), and optimizing fermentation parameters (Handa et al., 2019) could lead to more consistent and reproducible bioactive profiles. These advancements will not only improve the quality and efficacy of tempeh products but also support rigorous scientific studies and allow for validated health claims.

The sustainable production aspect of tempeh bioactive research also presents significant opportunities. As a low‐cost, health‐promoting, and environmentally friendly food, tempeh can contribute to sustainable food systems. By incorporating various beans (Tan et al., 2024), legumes (Erkan et al., 2020), and grains (Bento et al., 2021) in tempeh production, the diversity of bioactive compounds can be expanded, potentially leading to a broader range of health applications while also reducing reliance on animal‐based proteins.

When implementing processing technologies in the fabrication of soy‐based tempeh, it is important to prioritize several critical considerations to optimize product quality and functionality. First, the selection of appropriate processing technologies is paramount. Furthermore, precise control of processing parameters—such as temperature, pressure, and duration—is essential for ensuring consistency and reproducibility. Variations in these parameters can markedly influence the physicochemical properties and health benefits of the final product, necessitating rigorous monitoring and adjustment throughout the process. In addition, monitoring the levels of bioactive compounds during processing is also crucial. Ensuring that the concentrations of isoflavones, peptides, and other bioactive molecules meet the desired nutritional and functional criteria requires meticulous attention. Innovative technologies that enhance the bioavailability and bioactivity of these compounds should be prioritized to maximize the health benefits of the final product.

Moreover, safety and quality assurance protocols must be stringently implemented to prevent contamination and ensure microbial safety. Adherence to food safety standards through regular testing and validation is indispensable. Furthermore, the environmental impact of the processing technology should be considered. Sustainable practices and technologies that minimize waste and energy consumption should be prioritized to align with environmental conservation goals. By incorporating these recommendations, the processing and fabrication of soy‐based tempeh can be optimized, ensuring the production of a high‐quality, safe, and nutritionally beneficial product.

## 7 CONCLUSION

This review has discussed the complex characteristics of fermented soybean products, including tempeh, as a potential source of bioactive compounds for improving health. The bioconversion processes in the gastrointestinal tract, driven by enzymatic and microbial actions, significantly enhance the bioavailability and bioefficacy of soy bioactives. Key enzymes such as protease, leucine aminopeptidase, carboxypeptidase, glutaminase, γ‐glutamyl transferase, and amylase, along with microbial strains like *Rhizopus* spp., play a crucial role in transforming complex soy nutrients into bioavailable forms.

This review highlighted the extraction methods for isolating bioactives from fermented soy. These techniques have proven effective in increasing the yields of free fatty acids, vitamins, phytosterols, flavonoids, and polyphenols. For instance, fermentation has been shown to elevate the contents of phytosterols and total polyphenols significantly, demonstrating the process's potential to boost the nutritional profile of soy products.

Nevertheless, the findings from this review point toward significant opportunities and challenges in the research and development of tempeh and other fermented soy products. The limited global distribution of tempeh poses a challenge that can be addressed by increasing awareness and incorporating tempeh into diverse culinary traditions. Standardization of fermentation processes and leveraging advanced biotechnological tools will be essential for ensuring consistent bioactive profiles and reproducible health benefits.

In conclusion, fermented soy products like tempeh hold immense potential as next‐generation sources of bioactives for health optimization. Continued research and innovation in this field will not only advance our scientific understanding but also pave the way for the development of functional foods that promote health and sustainability on a global scale. The integration of tempeh bioactives into mainstream dietary practices, supported by rigorous scientific validation, could revolutionize the food industry and contribute to improved public health outcomes worldwide.

While significant advancements have been made in understanding the health benefits of fermented soy products, there remain substantial opportunities for further research. Future studies should focus on optimizing fermentation techniques to enhance the bioavailability and concentration of key bioactives, such as isoflavones and peptides. Additionally, clinical trials are needed to validate the health benefits of these bioactives in diverse populations. Investigating the interactions between bioactives from fermented soy products and the human gut microbiome also presents a promising area for understanding their role in health maintenance. These research efforts will help to establish evidence‐based guidelines for incorporating fermented soy products into health‐promoting dietary patterns.

## AUTHOR CONTRIBUTIONS

**Iskandar Azmy Harahap**: Conceptualization; data curation; funding acquisition; formal analysis; investigation; methodology; project administration; resources; validation; visualization; writing—original draft; writing—review and editing. **Joanna Suliburska**: Supervision; writing—review and editing. **Asli Can Karaca**: Writing—review and editing. **Esra Capanoglu**: Writing—review and editing. **Tuba Esatbeyoglu**: Supervision; writing—review and editing.

## CONFLICT OF INTEREST STATEMENT

The authors declare no conflicts of interest.
